# The impact of peer support on testing, linkage to and engagement in HIV care for people who inject drugs in Indonesia: qualitative perspectives from a community-led study

**DOI:** 10.1186/s12954-022-00595-8

**Published:** 2022-02-11

**Authors:** Arif Rachman Iryawan, Claudia Stoicescu, Faisyal Sjahrial, Kuntanto Nio, Alexa Dominich

**Affiliations:** 1 Rumah Cemara, Jalan Gegerkalong Girang, No. 52, Kota Bandung, 40154 West Java Indonesia; 2 School of Social Work, Columbia University, New York, USA; 3 Centre for Criminology, University of Oxford, Oxford, UK

**Keywords:** Peer support, HIV, ART, Adherence, Linkage to care, People who inject drugs

## Abstract

**Introduction:**

People who use, including those who inject, drugs in Indonesia are disproportionately affected by HIV, but tend to be diagnosed at a late stage of infection, delay initiation to and have poor rates of retention in antiretroviral treatment, resulting in high rates of morbidity and mortality. In addition to legal, policy and health system barriers, PWID may be hesitant to engage in HIV, treatment and care due to lack of knowledge, distrust of the health care system, and stigma related to their dual drug use and HIV status. Implementation of formal peer support initiatives may reduce provider- and individual-level barriers and increase testing, linkage to, and engagement in HIV care among people who use drugs.

**Methods:**

We conducted a community-led qualitative study to explore the impacts of peer support for people who inject drugs on HIV care access and engagement in Indonesia. Semi-structured, in-depth interviews were conducted with 20 participants in Jakarta and Bandung. Thematic analysis was used to explore how people who inject drugs living with HIV (PWID LHIV) (*n* = 8), peer support workers (*n* = 6), and service providers (*n* = 6) perceived peer support provision by non-governmental organisations.

**Results:**

Participants unanimously described peer support as beneficial. Peer support workers were widely credited with facilitating access to HIV testing, referral to care, uptake of and adherence to antiretroviral treatment, as well as sustaining engagement in care for PWID. Support mechanisms that facilitated positive peer experiences included provision of HIV knowledge and awareness, emotional support, help with navigating complex bureaucracy, developing trust in health care services, enhancing confidence and motivation, and supporting peers to navigate a wide range of health and social welfare services beyond HIV-related care.

**Conclusions:**

Findings indicate that peer support can enhance access to testing, linkage to, and engagement in HIV care for people who use drugs living with HIV in Indonesia. In a context of an ongoing HIV epidemic among people who use drugs, reduced funding and policy attention to HIV and harm reduction, there is an urgent need to prioritize peer support interventions to identify people who use drugs facing health risks and link them to appropriate services.

## Introduction

The scale-up of antiretroviral therapy (ART) has substantially reduced HIV-related morbidity and mortality worldwide [1]. ART has been widely endorsed as a highly effective strategy for both treatment and prevention [2, 3]. However, despite major strides towards treatment expansion in recent years, progress toward universal treatment access remains uneven. At the end of 2019, coverage of ART was only 60% in the Asia–Pacific region [1], which is 7% lower than the global average and well below the 95% target needed to end the AIDS pandemic [4].

Despite constituting a small proportion of the general population, key populations at higher risk of HIV transmission and their sexual partners, including men who have sex with men, sex workers, and people who inject drugs (PWID), account for 98% of new HIV infections in Asia–Pacific [1]. With an estimated 640,000 people living with HIV, Indonesia is one of few countries in the region where HIV prevalence is still increasing [5]. In the Indonesian context, HIV is concentrated among key populations, with people who inject drugs facing a high burden of HIV, at 29% [6].

The Indonesian government’s HIV response centres around a national HIV test-and-treat policy that integrates testing, referral to care, uptake of ART and adherence to treatment, in alignment with UNAIDS targets [4] and international guidelines for HIV testing [7] and treatment [2]. Furthermore, Indonesia has rolled out harm reduction interventions, including needle-syringe programmes, opioid agonist treatment and condom promotion to prevent HIV transmission among PWID for over 20 years [8, 9]. However, despite these policy and programmatic efforts, in 2018, only 51% of all PLHIV in Indonesia knew their status, of whom 17% had initiated ART [5].

The optimal public health benefits of HIV treatment-as-prevention can only be achieved when a high proportion of PLHIV are aware of their HIV serostatus, access and stay engaged in medical care, initiate ART at an early infection stage, adhere to antiretroviral regimens, and remain virally suppressed [2]. PWID are particularly likely to experience gaps at each juncture of the HIV care continuum [10]. In Indonesia, PWID tend to be diagnosed with HIV at a late stage of HIV infection, delay initiation to, and have poor rates of retention in ART and viral suppression [11]. A 2018 prospective cohort study conducted in Indonesia found that while a high proportion of PWID diagnosed with HIV were linked to care, only 67% of those diagnosed initiated ART [12]. Among those who started treatment, 55% were retained in care (had at least two outpatient visits at least 90 days apart after ART initiation) and only 22% of those retained in care returned for a viral load test 6 months after initiating treatment.

Late diagnosis and poor adherence to treatment are missed opportunities to prevent downstream transmission and AIDS-related deaths. Individuals who access care late also have higher rates of mortality and lower rates of treatment success once they initiate treatment [1]. Barriers to HIV medical care linkage, engagement and retention experienced by people who use drugs include punitive laws and drug-related policing practices that inhibit access to existing services by stoking fear of arrest and incarceration [13], health system barriers, including burdensome bureaucracy [14], limited competency and heavy workload of service providers, experienced and perceived stigma and poor treatment by healthcare providers [15], and lack of integration between ART and primary care [12].

The World Health Organization has emphasized the critical role of peer support in HIV prevention and in improving retention in care and viral suppression [2, 7]. Peer support programmes integrated into existing harm reduction, drug dependence and HIV services are effective in engaging, recruiting and supporting people who use drugs in HIV care by enhancing mutual trust, social support and knowledge, as well as reducing perceived stigma [16, 17]. ‘Peers’ are defined as individuals who share common characteristics or experiences, including, in this context, lived experiences of drug use and HIV, with those whom they support [18]. Peer support activities involve individuals with lived experience promoting access to and engagement in the HIV care continuum, including treatment adherence, as well as providing other types of support, from accompanying people who use drugs to access HIV testing, providing referrals to care, supporting individuals to adhere to medication through home visits, accompanying them to clinics for check-ups, and providing counselling interventions [2].

### Peer support in Indonesia

Between 2001 and 2009, Family Health International, through the U.S. Agency for International Development (USAID)-funded Aksi Stop AIDS programme, implemented the first outreach initiative for PWID that included integrated peer support and case management. The programme was run by 16 non-governmental organisations across six provinces, reaching approximately 6300 PWID [19]. Individuals reached by outreach workers through the programme typically underwent an individual risk assessment to assess their needs and guide referral to interventions aimed at maximizing health outcomes. Based on the risk assessment, a peer support worker followed up to facilitate and coordinate linkage to a broad range of healthcare services, including ART and social support. In this iteration of the programme, peer support workers and case managers had separate and complementary roles that, together, aimed to link people who inject drugs living with HIV (PWID LHIV) to a holistic range of health and support services and help them engage in HIV-related treatment and care.

Through the following decade, peer support and outreach diminished as part of responses to HIV and drug use, largely influenced by the changing funding landscape. In 2015, the withdrawal of funding for HIV programs in Indonesia by key international donors—primarily the American and Australian governments—had a detrimental impact on the national response to HIV and drug use [20]. The following year, the dissolution of the national coordinating body for cross-sectoral responses to HIV—the National AIDS Commission—left service providers without clear policy guidance in the national HIV response [13]. As result of these structural shifts, many governmental and non-governmental organisations were compelled to discontinue specific components of HIV programs for PWID, with some organisations ending almost all operations due to a lack of funds [21].

Presently, domestic funding for harm reduction programming in Indonesia prioritizes provision of sterile needles, as well as opioid agonist therapy and condoms, through Ministry of Health-run health centres (*puskesmas*), but excludes specific peer support interventions. Since comprehensive peer support does not benefit from dedicated domestic funding streams or explicit inclusion in national guidance on the HIV response, peer support interventions implemented ad hoc by a small number of organizations with funding from the Global Fund to Fight AIDS, Tuberculosis, and Malaria and from global HIV charity the Elton John AIDS Foundation.

While prior research has broadly examined the effects of peer support on linkage to prevention, care and treatment services in high-income settings, a better understanding of the impact of peer support on linkage to and engagement with HIV care among PWUD in geographically diverse and resource-limited settings is needed [22]. To our knowledge, this is the first community-led study to present a qualitative analysis of perceptions from people who inject drugs living with HIV (PWID LHIV), peer support workers, and service providers of the impacts of peer support for PWID on linkage to and engagement in HIV care in Indonesia.

## Methods

### Setting

The study was conducted in Indonesia’s capital, Jakarta, and Bandung, the capital of West Java province. The two study sites were selected on the basis of having the largest burden of HIV and greatest distribution of people who inject drugs in Indonesia [6, 23]. 61% and 28% of people who inject drugs living with HIV in Jakarta and Bandung, respectively, accessed ART [23].

### Research team

The design, aims, questions and procedures of this study were determined within a community-academic research collaboration guided by principles of community-based participatory research (CBPR) [24]. The study was led by peer experts from Rumah Cemara, a community-led organisation founded in 2003 to improve the quality of life of people using drugs and living with HIV, in collaboration with international academics. The research team comprised a research coordinator, two co-investigators, two interviewers and a research assistant, combining the expertise of lived experience of HIV and/or drug use, field outreach, programme delivery, and academic social science research.

### Participants

Between October and December 2019, we approached individuals across three respondent categories (i.e. peers/PWID LHIVs, peer support workers, and staff working for harm reduction service providers) to participate in the study. Sampling was purposive, seeking to capture heterogeneous experiences according to gender and age. Peers were eligible for the study based on the following criteria: injected drugs in the past year, lived with HIV, ever accessed ART, and participated in a peer support programme in the past year. Peer support workers were eligible to participate if they lived with HIV, ever injected drugs, and were employed as peer support workers for at least one year at the time of the study. PWID LHIV and peer support worker interviews were supplemented by interviews with staff working at government health centres and non-governmental organisations providing harm reduction services.

A total of 20 participants, 10 in Jakarta and 10 in Bandung, were interviewed for this study, comprising 12 males and eight females, and including eight PWID LHIV, six peer support workers, and six staff working with service providers across the two cities (see Table 1). Among the eight PWID LHIV who participated in a peer support programme as PWID LHIVs, 38% (three) were female. Mean age among the PWID LHIV was 40. PWID interviewed were aware of their positive HIV status for an average of 12 years, and all were ART-experienced.

**Table 1:** Summary of study participants

Respondent category	Jakarta		Bandung		Total
	Male	Female	Male	Female	
People who inject drugs living with HIV	2	2	3	1	8
Peer support workers	2	1	2	1	6
Service providers / programmatic staff	1	2	2	1	6
Total	5	5	7	3	20

### Procedures

Participants were recruited purposively to ensure diversity with respect to gender and age. We partnered with non-governmental, community-based organisations known to Rumah Cemara in the study areas that serve PWID through peer support programming. The opportunity to participate in the research study was announced by study staff to the organisations during their programme activities. Participating organisations in turn facilitated recruitment of HIV service providers, peer support workers, and PWID LHIVs. A small number of participants were recruited through harm reduction service providers where individuals were approached directly by study staff.

Individuals with lived experience of drug use and HIV were actively engaged at all stages of the research process. Two peer interviewers and a data manager were trained by senior researchers in qualitative interviewing, ethics, and data management and analysis.

All participants provided written informed consent. Consent forms were read and discussed verbally by the interviewers to ensure that participants had sufficient information to be able to provide informed consent regardless of literacy level. Interviews lasting 45–60 min were conducted in Bahasa Indonesia and/or Sundanese, a local language commonly spoken in Bandung, by trained interviewers, and were held in a private room or other private setting chosen by the participant.

Interviews followed standardized semi-structured guides (one for each of the three respondent categories), while also allowing flexibility to probe participant responses and explore emerging topics in greater detail. The guide for PWID LHIV and peer support workers elicited information about participants’ (a) history of HIV diagnosis and experience when initially engaging in the HIV medical care system; (b) perceived barriers and facilitators related to the health care system; and (c) experiences with peer support and case management. The guide for service providers focused on their assessment of the peer support programme in relation to linkage to care. The three semi-structured interview guides were developed by the community researchers, with input from academic researchers. Prior to field data collection, the interview guides were pilot tested with two respondents with similar characteristics as the intended participants, in order to enhance validity and appropriateness.

Participants received an incentive of Indonesian Rupiah IDR 350,000 (equivalent to ~ 25 USD) to cover their time and local transport costs. All names used are pseudonyms to maintain confidentiality. Ethical protocols for the study were approved by the ethics board at Atma Jaya University (ref. no. 1238/III/LPPM-PM.1xx0.05/09/2019).

### Analyses of qualitative data

Data analysis procedures, including coding and thematic analysis, were conducted jointly by the community and academic researchers. Interviews were audio recorded and transcribed verbatim with all personal identifiers removed.

A thematic analysis approach following Braun and Clarke’s six steps of qualitative analysis [25] was used to explore the data. Initially, two co-investigators read the interviews thoroughly to identify broad themes. PWID LHIV and peer support worker accounts were open coded, with coding driven by the central research questions and key issues highlighted by the participants, and then triangulated with experiences from service providers and programmatic staff. A working coding tree was developed to capture several initial themes related to perceptions of the peer support programme, to which the rest of the research team, including the interviewers, programme director, and data manager, were invited to contribute. Any discrepancies were discussed by the team and resolved. This process refined the coding structure, yielding a total of 19 codes. Transcripts were then coded by two of the co-authors independently and reviewed by the senior investigators to resolve any discrepancies.

All data that could be meaningfully categorized within the corresponding codes were transcribed verbatim into a summary table for each participant. The codes were then grouped into key overarching themes for each central question of interest by two co-investigators. Once the data were analysed, a consultation was convened to discuss and validate results with the peer research team, co-draft the manuscript, and devise strategies for dissemination of findings.

## Results

Findings from the qualitative analysis are organized into three main themes that provide insight into experiences of peer support: (1) impacts of peer support on linkage to and engagement in HIV care for PWID LHIV; and (2) mechanisms through which peer support operates to encourage linkage to and engagement in HIV care; (3) challenges and potential areas for improvement.

### Impact of peer support on testing, linkage to, and engagement in HIV care among PWID LHIV

Participants had overall positive experiences of peer support. Several key benefits of peer support on PWID engagement in the HIV care continuum were identified: facilitating linkage to HIV testing and referral to care, enhancing uptake of ART among newly-diagnosed individuals and those ‘lost to follow up’, and supporting ART adherence.

#### HIV testing and referral to care

Participants described peer support workers as their first link to HIV services, with many reporting that peer support workers enabled them to access HIV testing, subsequently enrol in treatment, and continue to engage with care. One individual described the essential role played by the peer worker in introducing him to HIV-related health services:

I know about peer support programme from the clinic, it’s part of the clinic’s services. It’s him [the peer worker] who told me, who outreached to me, who gave me information, helped and referred me. I got all that knowledge from peers, not from services. *(Male, PWID LHIV, 41, Bandung)*

Peer support workers commonly played a bridging role between PWID LHIVs and service providers, while maximizing the reach and quality of services. For many programme staff and service providers with limited capacity to pursue PWID LHIV who may be at risk of serious illness or otherwise lost to follow up, peer support workers played the critical role of contacting ‘hard to reach’ PWID LHIV and facilitating the continuation of their engagement with care.

The relationship between peer support workers and services certainly affects service access for PWID LHIVs, because when the relationship is good they can maximize their services with us. *(Male, Service provider, Bandung)*

Many individuals in the community feel helped when there is peer support. For example, they are PWID LHIVs who are difficult to contact and on average case managers helped. *(Female, NGO programme staff, Bandung)*

#### Uptake of ART and loss to follow up

Participants overwhelmingly described peer support as a helpful intervention in relation to improving the uptake of ART among PWID LHIV. PWID tend to avoid accessing ART until their health deteriorates [12]. This happens for many reasons, including due to misinformation about HIV and ART, fear of arrest based on their substance use, and stigma and discrimination [13]. The support provided by peer workers, including checking in on PWID LHIV through home visits, over the phone and text message and directly accompanying individuals to the clinic, is often the only link that marginalized PWID LHIV have to HIV service providers.

If they drop out of the treatment, we do a home visit, and then continue to accompany them to the service until they can access it again. *(Male, Peer support worker, 36, Jakarta)*

The challenge is usually that the PWID LHIVs we accompany are in a critical health condition. There are some who feel self-conscious, but we explain that if you don't seek treatment you may die soon. *(Female, Peer support worker, 31, Jakarta)*

Loss to follow-up from care among refers to individuals who initially accessed HIV testing services but did not return to the clinic to access follow up services for three months or longer [26]. Loss to follow up was described by participants as a critical issue in the community of people who use drugs in Indonesia. Peer support workers played a critical role in supporting individuals ‘lost to follow up’ to reconnect with health and support services and re-enrol on ART. Participants described how speaking with and accompanying someone mitigated that individual’s anxiety and fear regarding treatment and medical settings, and increased the likelihood that the PWID LHIV would agree to seek services at the clinic.

There are some PWID LHIVs who had not accessed antiretroviral treatment for almost six months. The peer support workers encouraged their peers to access treatment. [The outcome] is good, since many PWID LHIVs returned to access their treatment. *(Male, NGO programme staff, Bandung)*

Monitoring of loss to follow-up is actually a health service task, but if we don't wake them up they will not do anything. The health authorities themselves do not follow up. When I asked peers who stopped antiretroviral treatment, ‘Did any of the health services call or remind you or refer you back to treatment?’ the answer was no. *(Male, NGO programme staff, Bandung)*

Loss to follow up often occurs when people who use drugs are arrested and incarcerated. There is no ART provision in police detention in Indonesia, which can last several months [27]. ART provision in prisons is largely dependent on the willingness of family and friends to coordinate medication pickup from the clinic, liaise with prison officials, and deliver the medication to the prisoner. The family, together with peer support workers, must advocate on behalf of the PWID LHIV at each individual ART clinic and prison, in order to be able to deliver the medication to the prison. Peer support workers most often fill this crucial intermediary role, which enables incarcerated individuals to access ART by helping them procure the necessary referral letters and directly bringing them the medication.

There was a person using drugs and living with HIV in prison. Our peer support workers take their ARVs from the clinic and deliver it to the person in prison. *(Male, NGO programme staff, Bandung)*

Participants described how peer workers helped people who were newly released from prison to access ART by accompanying them through the administrative processes related to ART enrolment.

There are people who use drugs who have just been released from prison. Usually they don't have an identification documents or a health card. I once took care of administrative letters like that. *(Male, Peer support worker, 36, Jakarta)*

As soon as I was free from prison I immediately accessed my medicine at Pengayoman Hospital. The one who helped me was the peer support worker. *(Male, PWID LHIV, 36, Jakarta)*

#### Improved treatment adherence

Participants underscored the noticeable positive impact that peer support had on ART adherence. Several peers reported that peer support workers played a key role in how they engaged with treatment by regularly reminding them to take their medication through a phone call or text message, in person through regular home visits, and by providing ongoing encouragement and motivation when challenges arose.

He [peer support worker] always reminds me to be compliant, to set a phone reminder. *(Male, PWID LHIV, 31, Jakarta)*

Peer support is very influential in my ART medication adherence. When I am out with other peers around the time when I have to take my meds, they always remind me. This has already become a habit, a norm in the community of people who use drugs. *(Male, PWID LHIV, 41, Bandung)*

Picking up my medication has become easier. It’s easier to access services because my peer support worker is there. *(Male, PWID LHIV, 45, Bandung)*

One way in which peer support workers enhanced PWID LHIVs’ self-efficacy related to ART through their interactions was by embodying living examples of the success associated with treatment maintenance and continued engagement in care. Some explained how learning about or observing the treatment of their peers exemplified that treatment visibly improved the health and quality of life of PLHIV and that adherence was possible and realistic. One PWID LHIV explained how peer support workers’ treatment perseverance offered hope and contributed to his decision to initiate treatment. Emphasizing the shared lived experience of using drugs, living with HIV, and successfully adhering to treatment helped keep him engaged.

We just keep motivating them, so that they take their medicine regularly. I am an example showing that those who adhere to ART are healthy. *(Male, Peer support worker, 45, Bandung)*

### Support mechanisms in peer support

Participants identified a range of support mechanisms through which peer support was perceived to benefit PWID LHIV. These included: emotional support, improved HIV knowledge and awareness, assistance with navigating complex bureaucracy, increased trust in health care services, enhanced confidence and motivation, and enabling HIV, primary care and social support integration. These mechanisms were articulated in the context of improved linkage to and sustained engagement in HIV care.

#### Emotional support

Participants described how peer support workers provided advice, solidarity and a listening year when they needed encouragement to get tested, initiate ART, and adhere to the medication. The participants who had worked with a peer support worker to access HIV services reported feeling comforted knowing that the peer worker had undergone similar challenges. Peer support workers described the types of social support they offered, as well as the value of having shared similar experiences with clients:

When people just find out their [HIV] status, sometimes they don't accept it. We help them by providing information about HIV, we explain the pros and cons of not taking the medication. We also help them access BPJS [health and social security] and legal aid. *(Male, Peer support worker, 31, Jakarta)*

My experience is almost identical to his. I am also [HIV] positive, I'm also a drug user, so we end up wanting to share information with each other. From getting counselling to lab checks to starting medication, and sticking to taking it, to complaints related to drug use or medication problems, we bridge that divide so that the person does not feel alone. What he is experiencing I also experienced. *(Male, Peer support worker, 42, Bandung)*

This emotional support was credited with increasing PWID LHIV sense of commitment to the treatment regimen and motivation during periods of vulnerability or ill health. One participant explained that peer support enabled his continued adherence to treatment and engagement with care.

Once I got support from the peer worker, I was constantly being pushed not to give up, I had to commit. *(Male, PWID LHIV, 41, Bandung)*

Another participant described how talking to a peer support worker at a time when her emotional and physical health were deteriorating helped get her back on track to accessing healthcare.

It had been almost a year and a half that I'd been neglecting testing for HIV, I had depression, it had been a long time since I took care of myself. Finally after one of the peer workers talked to me, I asked him to take me to the clinic, and he said, let’s go. Before I got tested, I used to get ill often. *(Female, PWID LHIV, 45, Bandung)*

#### Enhanced HIV awareness and knowledge

People who use drugs in Indonesia receive information about HIV prevention, treatment and care from a variety of sources, including health workers and mass media. In many cases this information is dominated by inaccurate conceptions and myths based on populist morality [28], which stoke fear and play a key role in decisions against seeking health care [29]. Participants felt that information regarding HIV was easier to accept and comprehend when it came from peers rather than health providers or professional programme staff. This was informed by the shared experience of a drug-using background and the direct experience of taking ART. Participants described the accessible information provided by peer support workers as essential in equipping them with the knowledge and authority to dispel inaccurate information and fears about accessing HIV services and treatment, thus enabling them to make educated decisions.

In my community, it's easier to ask questions about HIV and AIDS. Asking the doctor is awkward, but from peers in the community I can get information easily. I tried to come to the government health centre (*puskesmas*), I asked about the HIV programme, but sometimes they don't understand me. *(Male, PWID LHIV, 41, Bandung)*

Participants described the language used by peers as accessible and relatable. Several participants emphasized the importance of the shared lived experience between peers and support workers, which reduced the power dynamics often present between professional health workers and peers.

This is why we need peer support workers. When peer support workers provide information, it is usually more effective than health workers who provide the same information. Information coming through peers is more acceptable to people who inject drugs. It gets through in a more meaningful way because the peer workers have experience with drug use, with taking ARVs. *(Male, NGO programme staff, Bandung)*

#### Navigating complex bureaucracy

Peers highlighted the value of peer support in helping them navigate complex bureaucratic procedures associated with accessing ART. PWID LHIVs noted that peer support workers provided practical assistance with accessing HIV testing, care and support, including by helping them to navigate administrative requirements, providing letters of referral, and physically accompanying PWID LHIVs to appointments.

I was assisted [a peer support worker] when I first accessed ARV. I didn’t understand the process or know what to do. Because there is a peer assistance programme, it makes access easier for us. *(Male, PWID LHIV, 45, Bandung)*

I assisted people who inject drugs and live with HIV to access several services that really helped improve their condition. There are people who don't have identify cards, documents. We are ready to help by communicating with service providers and other administrators on the person’s behalf. *(Female, Peer support worker, 31, Jakarta)*

#### Increased trust in health services

Participants described past negative experiences when interacting with staff at hospitals and clinics that provide HIV services. PWID LHIV often felt stigmatized based on perceived negative attitudes by care providers in relation to their drug use status, including observed excessive safety precautions taken by medical workers and denial of care. Women and gender-diverse people who use drugs faced added gender-specific challenges to accessing HIV services. One female peer described the limitations of service providers in addressing her reproductive health needs while she was actively using drugs. Another participant explained that LGBTQ + individuals who use drugs often faced double stigma related both to their sexual identity and their substance use. These experiences created negative expectations about accessing HIV testing and initiating treatment. As a result, some participants postponed initiating ART or returning to the clinic for check-ups, and many lost trust in medical services.

I empathize with folks who are still active (using drugs). If they access health care, ARVs, they will surely be asked all kinds of questions that give a clear impression that the doctor doesn't believe that they will adhere to medication. *(Female, NGO programme staff, Bandung)*

There are still service providers who, if they see someone who is [HIV] positive, thin, emaciated, they are discriminatory. As soon as we came in, they immediately put on gloves, masks. *(Male, Peer support worker, 36, Jakarta)*

Health services for sexual and reproductive health are not working well. The stigma is still there. *(Female, NGO programme staff, Bandung)*

Respondents pointed out that peer support workers may reduce the effects of stigma and discrimination from medical staff and help restore clients’ trust in the medical system. PWID LHIVs explained that having positive experiences with healthcare services when they were accompanied by peer support workers encouraged them to stay engaged in care.

The role of peer support is to motivate us to trust in health services, more or less, yes. Peer support is quite helpful. We don't know where to go otherwise, but if there is a peer support worker, it’s alright. *(Male, PWID LHIV, 36, Jakarta)*

#### Confidence and motivation

As a result of receiving peer support, several PWID LHIVs described feeling more motivated and confident accessing care and adhering to medication. The process through which peer support appeared to impact motivation may be through the development of support networks, the promotion of optimism, and provision of practical support with navigating bureaucratic procedures and discriminatory health service providers.

With peer support I became more confident. Accessing ARV means a lifetime commitment. *(Male, PWID LHIV, 41, Bandung)*

Peer support has a lot of impacts, one of which is that PWID LHIVs are more aware, they begin to take their medication as prescribed and go for their lab tests regularly, which means that they value themselves and want to know about their current health status. *(Male, Peer support worker, 42, Bandung)*

#### Service integration

Peer support workers, whose formal role is primarily intended to support newly-diagnosed individuals to access and remain on ART, in practice assisted peers with a much wider range of ancillary services, including referrals to primary care, psychosocial and counselling support, accessing opioid agonist treatment, health and harm reduction services post-release from prison, accessing social insurance, applying for identification documents, and legal aid services, among many others.

If the client asks for our help, for example, to help mediate disclosure or a conflict between them and their families, we are ready to help them. *(Female, Peer support worker, 31, Jakarta)*

If clients have a legal problem, I help to at least provide information and guide them through the process. *(Male, Peer support worker, 36, Jakarta)*

One individual described his experience of receiving support from a peer support worker for an ongoing drug-related legal case. The peer worker provided letters of referral and accompanied him through complex bureaucratic hurdles, including securing a medical assessment in order to confirm his eligibility for diversion to drug dependence treatment. The client attributed the peer support worker’s assistance with ultimately avoiding incarceration.

The peer support worker has a good relationship with service providers, which makes access easier. He always accompanies me not only in relation to ART, but also on how to access methadone, and for personal and legal matters. In terms of legal matters, he helped me avoid prison: he wrote a letter, [helped with] my medical assessment, and sent supporting documents to the police. *(Male, PWID LHIV, 36, Jakarta)*

Several participants attributed improvements in their health status, as well as better quality of life, to receiving support from peer workers, who, for some individuals, represented their only link to health services:

They help people who have just found out their HIV status. Apart from that access, they also give them other types of support, like counselling and easy, quick information. They react quick, if you say, ‘please reach out here, someone is sick, they need to be reached.’ If the peer worker does not connect with them and bring them to services, then they remain sick. *(Female, PWID LHIV, 45, Bandung)*

The impact is that the [client’s] general health is better than before we assisted them. For example, one person who had stopped taking his medication, and was so sick that he could not walk and was in a wheelchair, is now [back on treatment] able to walk and go about their activities. *(Female, Peer support worker, 31, Jakarta)*

### Challenges and areas for improvement

#### Punitive drug law enforcement

Indonesia has some of the harshest drug laws in Asia [27]. Punitive drug law enforcement practices, including street-level arrests for personal drug use and possession, create an environment of fear that dissuades people who use drugs from seeking care [30]. One participant noted that the ambiguity of the drug law, which fails to clearly differentiate between those who use drugs and those who engage in drug trafficking, leads to many people who use drugs who require health and social support to instead be incarcerated.

People who use drugs are still getting arrested […] There is no such thing as users being diverted to rehab. In practice, it is not as straight-forward as national authorities say it is. *(Female, Programme staff, Bandung)*

#### Declining quality of care with reduced funding

Due to decreasing funding for HIV prevention and harm reduction, the peer support component of the national HIV response for people who use drugs has been significantly reduced in recent years. As a result, peer support programmes operating in Indonesia at the time of this study were often compelled to combine harm reduction outreach, education, support and case management. Participants felt that the need to fulfil multiple roles had tangible negative effects on the quality of peer support services. External donors, participants explained, often applied unrealistic targets that require peer support workers to assist a minimum number of clients each month. At the same time, diminished funding from a smaller number of donors has led to a reduced number of peer workers handling increasing caseloads. Participants described how these changes led to overwhelmed peer workers who are no longer able to assist clients with all of their needs:

Now it’s about chasing targets, not about quality. Because we are confronted with these targets, we are forced to abandon the quality. Peer-led counseling that used to take 2-3 hours is now very brief or often does not take place at all. *(Male, Peer support worker, 42, Bandung)*

Since most of our funding comes from external donors, they don’t look at service quality any longer. They are only concerned with quantity, they are only concerned with numbers […] Our outreach target is 1,400 PLHIV – it’s too high. *(Male, Peer support worker, 31, Jakarta)*

One NGO programme staff reflected on another negative impact of client quotas that do not appear to reflect the reality on the ground. Because harm reduction materials and continuing funding are tied to reaching numerical targets, some peer support workers and service providers feel compelled to double count clients if they cannot meet their monthly quota of PWID PLHIV linked to care.

Does it seem rational, if for example, one month we get one or two positive PWID, but according to our target we have to get ten? The effect is that we will count past clients and report double the data. *(Male, NGO programme staff, Bandung)*

One peer support worker described becoming ill due to overworking in an attempt to reach his “quota”:

I was so focused on chasing the target, I became ill, my eyes were all yellow. *(Male, Peer support worker, 36, Jakarta)*

A further consequence of decreased funding for peer support interventions was a lack of investment in adequate compensation and professional capacity building for peer support workers. With declining donor funding, investment in harm reduction materials and peer workers’ capacity needs appears to have dwindled. This includes a perceived reduction in the availability of condoms and sterile needle-syringes typically distributed by peer support workers when conducting outreach and assisting peers.

In the past, from 2006 to 2016, peer workers distributed a full range of harm reduction equipment: there were condoms, there were syringes, information, education, and communication (IEC) materials. But currently peer workers rarely have such materials. *(Male, Peer support worker, 42, Bandung)*

In terms of HIV prevention, the supply of needles is now decreasing. In the past, the needles were of the best quality, but now, we get a lot of expired needles, alcohol swabs that are already dry... *(Male, NGO programme staff, Bandung)*

### Improvements needed to sustain benefits of peer support

Respondents underscored the need to invest in the capacity of peers so that they can effectively support the community of people who use drugs. Suggested improvements included: recruiting additional peer support workers, meaningfully involving peer workders in the design and evaluation of HIV-related services, providing regular capacity building on HIV/AIDS and ART, and equipping peers with harm reduction materials including low dead space needles, updated information, education, and communication (IEC) materials, and naloxone to reverse opioid overdose.

The capacity building of the peer support workers must be increased, and they must be more meaningfully involved My hope is to not have targets, but focus on quality. On how we can better support sharing of information, IEC. (Male, Peer support worker, 42, Bandung).

One peer support worker proposed that the government increase political commitment and investment in HIV prevention and care targeted toward people who use drugs, including peer support work. This includes adequately compensating peer support employment and recognising peer support workers as essential programmatic staff, rather than temporary, casual or part-time workers.

The government should be more supportive, yes, so that us NGOs don’t depend on foreign donor agencies. *(Female, Peer support worker, 31, Jakarta)*

Participants emphasized the need for donors and programme managers to be more responsive to co-morbidities such as hepatitis C (HCV) and to the overdose crisis amongst PWUD, and adequately equip peer outreach workers as first responders. Specifically, participants perceived a recent increase in overdose deaths. The illegality of community distribution of naloxone, a drug that reverses the effects of opioid overdose, was noted as a significant barrier to an effective overdose response in Indonesia. One peer support worker described the need for peer outreach workers to be trained in overdose prevention, including administering naloxone:

Many of our peers have overdosed, and for now the outreach and peer support workers are not equipped with the appropriate tools. I mean, the first response that we can provide is not enough, unless we have naloxone which can neutralize the effects of overdose. *(Male, Peer support worker, 42, Bandung)*

Participants additionally emphasized the need to enhance peer support workers’ knowledge and capacity to support testing and treatment for HCV, which affects 51.5% of PWID in the country [31].

As soon as we began to feel safe with HIV cases among PWID decreasing, it turns out that HCV has been increasing. Our finding is HCV is quite high but there is no action yet. *(Male, NGO programme staff, Bandung)*

## Discussion

This study presented qualitative perspectives from peers, peer support workers, and HIV service providers on a peer support programme operating in Indonesia. Participants described multiple benefits of peer support interventions for people who inject drugs, including facilitating HIV testing access, treatment initiation and adherence and supporting sustained engagement in HIV care. These positive outcomes were enabled by key support mechanisms, including validation and emotional support resulting from shared lived experience between peers and peer support workers; normalization of health-seeking behaviours and ARV treatment experiences; clarification of misconceptions and provision of factual, accessible HIV information; alleviation of anxieties and fears related to accessing testing and treatment; and practical assistance navigating complex bureaucratic structures beyond HIV-related services to meet individuals’ broader health and social welfare needs. People who inject drugs reported that these factors helped them regain trust in health care services, develop confidence, and stay motivated and engaged in HIV care.

Previous systematic review evidence indicates that peer support and education are effective in promoting HIV testing and retention in care [32] and reducing unsafe injecting and sexual risk behaviours among key populations [33]. However, few studies to date have examined the utility of peer support in relation to HIV treatment cascade outcomes in people who use drugs in Asian contexts. No studies have explored this topic in Indonesia, despite more than a decade of peer support programme implementation in the country. Our findings provide qualitative evidence suggesting that peer support workers have the potential to maximise the impact of treatment-for-prevention in Indonesia by improving PWID engagement in HIV care. Peer support workers, who have lived experience of drug use and ART treatment, are uniquely situated to address psychosocial challenges faced by PWID in initiating and continuing HIV care, including by playing a mediating role between community members and service providers and boosting peer motivation by exemplifying health-seeking and pro-social behaviours. Previous studies have shown that formalized support from peers with shared stigmatized identities, psychological and health challenges is particularly helpful [17], given the substantial barriers to treatment uptake posed by HIV- and drug use-related stigma [34].

It is important to note that many of the benefits of peer support noted by participants in this study may also be experienced in informal community settings where peers share knowledge and lived experience [18]. However, the benefits of formal peer support programmes for people who use drugs that are officially recognised and funded as part of policies and strategies relating to the national HIV response, should not be undervalued. Based on experiences in the United States [17, 35] and Australia [36, 37], having a structure in place that allows treatment- and drug use-experienced peers to interact with and provide guidance to individuals in the community with formal backing from authorities and service providers are essential for facilitating the benefits of peer support found in our research. Formalized, adequately resourced peer support programmes inherently provide the supportive institutional framework that can facilitate effective HIV service linkage and support to underserved communities of people who use drugs.

Our findings indicate that peer workers in Indonesia are not equipped to engage effectively in facilitating support and linkage to care without the necessary resources to keep them informed of the latest HIV, treatment, and harm reduction developments, supplied with key harm reduction materials and adequately paid. Such resources also include ongoing training in communication and capacity building on addressing emerging community needs. Specifically, participants noted that assistance provided by peer support workers often extended far beyond their official roles pertaining to facilitating HIV services engagement among people who inject drugs to include acting as first responders to overdose incidents, providing counselling on personal and family issues, and assisting peers with legal aid, health insurance and procuring identification documents. This finding is consistent with previous research in high-income settings showing that peer support workers are often compelled to take on expanded roles, which in turn takes a substantial mental health toll on them and is typically not financially compensated [38]. While our study did not specifically explore the impacts of peer support on peer support workers, one peer support worker reported becoming ill and interrupting medication due to overwork, while several others remarked that training, human resources, and financial support for the peer support workers in Indonesia have decreased substantially in recent years. Alongside shrinking funding, participants noted an increase in target-driven outcomes, which they felt compromise the range and quality of the assistance that peer workers can provide to their communities, and may undermine the proven benefits of peer support interventions on engaging and retaining PWID in HIV care [18]. Ensuring the sustainability and continued benefits of peer support for the HIV response for people who use drugs in Indonesia requires a re-focusing of programmatic priorities toward equipping peer support workers to deliver quality guidance to their peers, while supported by adequate funding and compensation, explicit inclusion in national guidance, and formal recognition of the value of the broad range of roles performed by peer support workers through standardized operating procedures and guidelines. Underscoring these suggested improvements is the need to meaningfully engage peers and peer support workers in programmatic and policy decisions.

A key strength of our study was our community-led, participatory approach. By creating partnerships between researchers and the people whom the research is ultimately meant to impact, this study sought to combine the expertise of lived experience, advocacy and programming with academic social science research, in order to promote better use of research evidence and build research capacity in affected communities [39]. This approach allowed us to bring the insights and experiences of peers into all aspects of the research process, while allowing community researchers learn with academic professionals and develop the confidence and critical skills to design, manage and carry out research.

A limitation of the study is the inability of generalize findings to other populations of people who use drugs across Indonesia and other international settings. Future research would benefit from additional insights from quantitative evaluations of peer support interventions for diverse communities of people who use drugs in Indonesia, which could inform the agenda for national and international donor priority setting for accelerating the HIV response. For example, recent increases in the use of crystal methamphetamine in Indonesia have been associated with increased risk of blood borne virus transmission [40]. While gaps in existing harm reduction service provision for people who use methamphetamines are concerning [41], emerging evidence suggests that peer support in the form of brief peer-led mental health interventions and provision of safer-smoking kits improve linkage to health services for people who use crystal methamphetamines [42]. These are promising areas that require further investigation. 

## Conclusion

Findings from this study indicate that formalized peer support programmes may have an essential role to play in optimizing HIV prevention and treatment outcomes among communities of people who use drugs. While policy, health system and site-level barriers for people who use drugs to access and engage in the HIV care continuum [12, 13], peer support offers a viable and acceptable solution to enhance support systems for access to testing, treatment initiation and adherence and reduce common psychosocial and community-level barriers, thus improving HIV outcomes for this underserved population.

## Data Availability

Data sharing is not applicable to this article because individual privacy could be compromised and because the participants are part of a marginalized and stigmatized group requiring protection.
